# “Mikroskopischer Farbatlas pflanzlicher Drogen”

**DOI:** 10.3797/scipharm.br-10-04

**Published:** 2010-09-30

**Authors:** Liselotte Krenn

**Affiliations:** Department of Pharmacognosy, University of Vienna, Austria

Teaching of microscopy in the pharmacy curricula in Europe has almost vanished, although adulterations and mistakes in identification of herbal substances have caused several problems during the last decade. Thus, profound knowledge in the macroscopic and microscopic authentication of herbal drugs is an indispensable prerequisite in quality control.

This book provides an excellent overview over macroscopical and much more detailed over microscopical features of more than 140 herbal substances used in European phytotherapy. Those drugs with existing HMPC-, WHO- and ESCOP-monographs have been prioritized. In each of the presented monographs the precise botanical name of the respective plant (under inclusion of recent systematic findings), a photograph of macroscopic characteristics, a short compilation of the most important compounds and the use of the herbal substance is given. Additionally, six clear coloured charts of the most important microscopical characteristics are shown and described in detail. The drugs are arranged according to the plant part used (leaves, herbs, flowers, fruits, seeds, roots, barks and rhizomes) and presented in alphabetical order based on the German names. Finally, some instructions for investigations under use of colouring reagents for specific compounds, a glossar and charts explaining the structure of several plant organs follow. This book is highly recommended to all interested pharmacists whether in public pharmacies or in quality control of herbal substances in industry as well as especially for students, because it provides a detailed insight into this more and more neglected field. The in-depth microscopical part makes the book a valuable teaching tool and excellently suitable for self-learning.

